# Safe Discharge Needs Following Emergency Care for Intimate Partner Violence, Sexual Assault, and Sex Trafficking

**DOI:** 10.5811/westjem.59072

**Published:** 2023-05-02

**Authors:** Michael J. Clery, Eudora Olsen, Hannah Marcovitch, Harrison Goodall, Jasmine Gentry, Matthew A. Wheatley, Nichelle Jackson, Dabney P. Evans

**Affiliations:** * Emory University School of Medicine, Department of Emergency Medicine, Atlanta, Georgia; † Emory University School of Medicine, Atlanta, Georgia; ‡ Emory University School of Medicine, Grady Health System, Emergency Care Center, Atlanta, Georgia; § Emory University Rollins School of Public Health, Atlanta, Georgia

## Abstract

**Introduction:**

For survivors of gender-based violence (GBV) seeking care in hospital emergency departments (ED) the need for medical care and safe discharge is acute.

**Methods:**

In this study we evaluated safe discharge needs of GBV survivors following hospital-based care at a public hospital in Atlanta, GA, in 2019 and between April 1, 2020–September 30, 2021, using both retrospective chart review and evaluation of a novel clinical observation protocol for safe discharge planning.

**Results:**

Of 245 unique encounters, only 60% of patients experiencing intimate partner violence (IPV) were discharged with a safe plan and only 6% were discharged to shelters. This hospital instituted an ED observation unit (EDOU) to support GBV survivors with safe disposition. Then, through the EDOU protocol, 70.7% were able to achieve safe disposition, with 33% discharged to a family/friend and 31% discharged to a shelter.

**Conclusion:**

Safe disposition following experience or disclosure of IPV and GBV in the ED is difficult, and social work staff have limited bandwidth to assist with navigation of accessing community-based resources. Through an average 24.3 hours of an extended ED observation protocol, 70% of patients were able to achieve a safe disposition. The EDOU supportive protocol substantially increased the proportion of the GBV survivors who experienced a safe discharge.

## INTRODUCTION

Intimate partner violence (IPV), sexual assault, and sex trafficking are forms of gender-based violence (GBV), which results in preventable morbidity and mortality. In the US, one in five women experience severe physical violence from an intimate partner during their lifetime; likewise, one in five women have experienced rape with even more experiencing any form of sexual violence.^1^ While human trafficking is especially hard to measure it is known to share the same risk and consequences as IPV and sexual violence.^2^ Since the onset of the coronavirus 2019 (COVID-19) pandemic, GBV has increased in the US and globally.^3^–^8^ Gender-based violence describes violence toward an individual based on their gender; for our purposes we use the term to reference three forms of GBV—IPV, sexual violence, and sex trafficking—as these were the specific forms of violence measured within our study setting.

With GBV survivors seeking care in hospital emergency departments (ED) the need for survivor identification, medical care, and safe discharge is acute. Many studies have sought to measure the presence of GBV cases in hospital EDs, but even before the pandemic accurate quantitative estimates were challenging to gather given stigma and survivor hesitancy to disclose experiences of abuse, violence, and exploitation.^11^–^13^ In addition to the barriers faced by survivors in seeking care, ED staff often face significant challenges in assessment and treatment of patients experiencing violence due to time constraints, insufficient training, and lack of systematic processes, including a process for referral to further services.^13^,^14^

Constraints on time in a fast-paced ED setting are barriers to the identification of GBV survivors.^14^,^16^ While screening can lead to survivor identification and help to reduce recurrent hospital visits, it also has a number of limitations, namely that it does not necessarily promote referral or linkage to community services.^14^,^17^–^19^ There is an urgent need to develop models for referral and community support services after survivors leave the ED. One study that examined the patterns of abuse reoccurrence after severe injury presence in the ED due to IPV found that only 19% of patients were referred to “advocacy,” regardless of severity of injury or likelihood of IPV reoccurrence.^20^ Additionally, these same patients were likely to have experienced severe violence and were at high risk for IPV reoccurrence and/or death. Successful efforts to improve GBV care and referral to services in EDs have included standardizing forms/assessment tools, funding specialized nurses, staff training, and building electronic health records systems (EHR) to detect previous incidences of IPV.^13^,^21^

Social conditions and well-intended pandemic mitigation tactics exacerbated GBV including increased likelihood of abuse and exploitation, and loss of access to social supports and community resources.^7^,^9^,^10^ As the pandemic began in Spring 2020 ED clinicians at a large, safety net hospital in Atlanta GA, observed increased difficulty attaining safe discharge plans, including connections to community resources, for survivors of GBV. Our goal in this study was to assess the needs of survivors of IPV, sexual assault, and sex trafficking to secure a safe discharge plan following hospital-based care.

## METHODS

### Design

After receiving medical care, individuals who are clinically assessed as having experienced violence, have disclosed experiences of violence, or screen positively for IPV or sex trafficking are routinely referred to ED-based social worker to identify their need for social support services. Survivors of GBV presenting to EDs often rely on social workers to help identify a safe disposition plan. We examined the hospital’s ED social work encounters during 2019. This included both review of a social work patient log and associated EHR charts. After assessing the distribution of social work encounters, we conducted a chart review on patients presenting to the ED in 2019 who reported IPV, sexual assault, or sex trafficking to determine disposition after their ED encounter.

**Population Health Research Capsule**

What do we already know about this issue?

*Gender-based violence (GBV) such as intimate partner violence and sex trafficking is prevalent; emergency department (ED) patients often require assistance to access a safe discharge plan*.

What was the research question?

*How frequently are ED patients unable to access a safe discharge, and does a novel ED observation protocol improve safe discharge*

What was the major finding of the study?

*Through a novel ED observation protocol, 70% of the patients who did not have a safe discharge plan were able to achieve one*.

How does this improve population health?

*Understanding facilitators of safe discharge plans such as an ED observation protocol allows EDs to support secondary prevention of re-injury or another form of GBV*.

In addition, beginning in April 2020, patients identified as survivors of IPV or sex trafficking with no safe discharge location and a desire for placement were assisted by an ED social worker to contact local shelters. If no bed was available, the patient was placed in the emergency department observation unit (EDOU) for assistance in further contacting local shelters, arranging transport to out-of-state family, and/or contacting supportive family or friends. A separate chart review was performed for the patients placed on the EDOU supportive care protocol over the first 18 months (April 1, 2020–September 30, 2021) to understand the feasibility of implementation and any barriers experienced in safe patient disposition.

### Ethics

We obtained social work data through the hospital quality/performance improvement data request form process in compliance with the hospital data-use agreement. The Emory University Institutional Review Board determined that based on its nature as quality improvement this study did not meet the criteria for human subjects research and was exempt from review.

### Data Management and Analysis

#### Social Worker Chart Review

Social workers in the ED record daily patient encounters in a shared Excel file (Microsoft Corporation, Redmond, WA) that is organized by month. The monthly ED social work records were combined into a single Excel file collating data from January 1–December 31, 2019. To assess the distribution of social work effort, we first sorted data based on the “problem” variable, an open-ended variable without coding or preset categorization. The entire dataset was categorized to the greatest extent based on the open-ended variable entry. Of the entries, we were able to categorize 69% into 11 service issues: traumatic injury resuscitation; medical resuscitation; transportation; family contact; housing/shelter; substance use disorder; IPV; sexual assault; human trafficking; non-partner abuse (violence perpetrated by someone who is not identified as a “partner” of the victim); and physical assault. The remaining 31% did not fall into one of these predetermined categories and were thus marked as “other.”

To identify and verify all 2019 encounters related to GBV, data cleaning began with an examination of the “problem” field. Encounters unrelated to IPV, sexual assault, or sex trafficking were excluded; some unspecified encounters that remained as the recorded problems were non-specific in nature. Next, we deleted duplicate entries (entries for the same patient encounter on the same date), leaving 2,201 charts for comprehensive review.

All EHR chart clinical notes were reviewed to confirm the “problem” category, resources provided, and ultimate disposition from the ED. We chose problem categories (domestic violence, sexual assault, human trafficking, shelter, financial resource counseling, manage police contact, other, unknown, unable to review) and disposition categories from standardized options (discharge to self, discharge to friend/family, discharge to home, discharge to domestic violence/human trafficking shelter, discharge to homeless shelter, admit, psychiatric admission, eloped), respectively. “Discharge to self” reflected being discharged without an identified home or shelter and typically reflected a patient being undomiciled without an available shelter bed identified; eloped referred to those individuals who left of their own accord without receiving further care. After chart review, identifiable information was removed and a unique identifier assigned to each entry. We ran basic descriptive statistics using Excel to assess the social work “problem” and disposition across survivors of IPV, sexual assault and sex trafficking.

#### ED Observation Unit Protocol Chart Review

We reviewed EDOU patient records for the “general observation” protocol between April 1, 2020–September 30, 2021, and found that 17 patients had received the observation protocol for safe disposition support related to IPV, sex trafficking, or sexual assault. We performed a chart review for the related clinical encounter for each patient identified and reviewed clinician and social work notes from the encounter. Patient demographics, length of stay, barriers to discharge, and whether the encounter occurred on a weekend were recorded. Dispositions were chosen from standardized options (discharge to self, discharge to friend/family, discharge to home, discharge to domestic violence/human trafficking shelter, discharge to homeless shelter, admit, psychiatric admission, eloped). After chart review, we used Excel to run basic descriptive statistics to assess barriers to discharge and disposition across survivors of IPV, sexual assault, and sex trafficking.

## RESULTS

**Figure 1:**
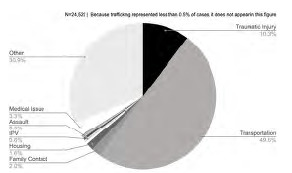
Social work effort by problem at a public hospital emergency department in Atlanta, Georgia (2019).

In the ED, social workers were staffed 24 hours per day, seven days per week, and provided support in 24,522 patient encounters in 2019. Nearly 50% (12,164) of entries were related to arranging transportation, demonstrating the overwhelming burden of transportation logistics that is borne by the social work team in this ED. These tasks include checking insurance coverage, contacting medical transportation, and arranging transportation with hospital-based transportation services. Other problem areas of note included responding to traumatic injury resuscitations (10.3%) and medical resuscitations (3.3%), assisting with family contact (2.0%;), and responding to housing needs (1.6%398). Notably, 138 of the ED social worker encounters were explicitly related to IPV (0.6%), 50 to sexual assault (0.2%), and 47 to sex trafficking (0.2%) (See Figure 1).

Through chart review we identified 245 unique social worker encounters for IPV in 2019. Almost all the entries reflect separate individuals, although 24 individuals were treated for IPV more than once in the year. We found that 97 IPV patients (40%) were discharged with no identified safe shelter, essentially being discharged to the street (Table 1). The proportion of patients discharged without a safe plan or shelter increased across later shifts (37% between 7 am–3 pm; 40% from 3 pm –11 pm; and 44% from 11 pm –7 am). We found that 69 patients (≈28%) were discharged to a family member or friend with whom they felt safe, and 49 (20%) felt safe returning to their own home with notes often reflecting the assailant had been arrested or was not living in the same home. Only 14 patients (6%) were discharged to a domestic violence shelter. Social work notes typically reflected assisting the patient in calling one or more of the local domestic violence shelters and being told there were no beds available. Thirteen patients (≈5%) were admitted to the hospital for additional medical care.

During 2019, 45 recorded social work encounters followed a positive screen for sex trafficking, of which 19 (42%) were identified as likely having experience of sex trafficking. Nine patients (≈50%) who were identified with likely experience of trafficking were “discharged to self” with no safe shelter or community organization assistance (Table 2). Four patients (≈20%) were discharged either to home (two) or with family or friends (two) who were reported to be safe. Two patients (≈10%) were discharged to a human trafficking or domestic violence shelter, and two (10%) were discharged to a general homeless shelter.

There were 94 social work encounters for sexual assault in 2019 (Table 3). A total of 53 (56%) sexual assault survivors were recorded as discharged to self; however, the disposition was less reliably recorded for victims of sexual assault, likely reflecting lack of explicit disposition planning unless sexual assault occurred in their residence. Among sexual assault survivors, 30 (32%)were experiencing homelessness in a way that was associated with the assault. This included individuals who accepted invitations for shelter or use of amenities due to experiencing homelessness and subsequently being sexually assaulted, as well as individuals who were victimized while homeless and traveling or sleeping in a public space.

In response to the COVID-19 pandemic, a protocol for extended observation in the EDOU was established to assist with the safe discharge for survivors of GBV. Over 18 months (April 1, 2020–September 30, 2021) 35 survivors of IPV (58%), 10 survivors of sex trafficking, and 10 of non-partner violence were placed on the EDOU supportive care protocol. All identified as female, except for one who identified as transgender female and one male. The average length of stay in the EDOU was 24.3 hours. Among cases placed on the EDOU supportive care protocol 41 patients (70.7%) were able to achieve safe disposition. Of those on the protocol for IPV, 29% had been previously treated for IPV within the prior year.

Eighteen patients (≈31%) who participated in the EDOU supportive care protocol were ultimately discharged to a shelter and 19 (33%) were discharged to a family or friend they were able to contact during the extended observation, while 17 (29%) were ultimately “discharged to self” with recommendations to pursue local homeless shelter services (Table 4). Patients were relatively less likely to be discharged to a shelter bed on a weekend (40% weekday; 25% weekend). The primary barrier to safe disposition for 28 survivors of IPV and sex trafficking (62.2%) was shelter bed availability, but for four patients (9%) transportation to shelter and for one patient (2%) substance use disorder were also noted as barriers to disposition.

## DISCUSSION

We examined social work encounters at a large, safety-net hospital in metropolitan Atlanta during 2019 to understand the safe discharge needs of survivors of IPV, sexual assault and sex trafficking. Our finding that over 50% of recorded encounters were related to arranging transportation demonstrates the overwhelming burden of transportation logistics that is borne by the ED social work team. These appear to be tasks that may be undertaken by a clerk rather than licensed social workers with specialized clinical skills. Health systems could consider task-shifting logistical responsibilities from clinicians to clerical or support staff and partnering with municipal transit authorities as well as private ride-share organizations to provide vouchers to those in need.

Safe housing was a major unmet need among GBV survivors. We found that 40% of IPV survivors and 47% of sex trafficking survivors were discharged without confirmed safe housing. One third (32%) of sexual assaults in this analysis were directly related to the experience of homelessness. All survivors of violence would benefit from safe dispositions planning; for survivors of sexual assault, the hospital may leverage standard Sexual Assault Forensic Exam protocols so that all survivors are evaluated for a safe discharge plan. Survivors of sex trafficking would benefit from increased coordination between hospital-based care and community-based anti-trafficking organizations that could provide early wraparound services and emergency shelter. Survivors of IPV would benefit from increased bed capacity at IPV-specific shelters, while all survivors would benefit from increased temporary shelter access.

The EDOU supportive care protocol was created in response to the spike in domestic crimes in Atlanta at the outset of the COVID-19 pandemic during the time when stay-at-home orders were in effect and domestic violence crimes increased weekly while local shelters operated with limited capacity.^22^ The EDOU supportive care protocol was designed to support the most isolated patients experiencing violence who do not have an immediate support network to offer safe shelter; the protocol allows for up to 48 hours of social work assistance in shelter placement for victims of IPV and sex trafficking and included collaboration with a local IPV shelter manager to build the capacity and enhance contacts for ED social work staff. While the EDOU supportive care protocol was borne out of the pandemic, it has continued to serve as a critical bridge between the most isolated patients experiencing IPV and sex trafficking and needed shelter and support resources. The EDOU supportive protocol substantially increased the proportion of GBV survivors who experienced safe discharge through increased time to access community- and personal-support networks. In the future this program should be more rigorously evaluated to determine its effect on improved hospital-based care and uptake of community-based social services.

This initial review of the EDOU supportive care protocol raises specific concerns for the safe-discharge needs of chronically undomiciled survivors. For undomiciled IPV survivors, traditional IPV social support services may be especially challenging to access. In such cases, while IPV may not displace an undomiciled individual from their home, it may disrupt a relationship that is protective against other forms of violence, or it may otherwise be difficult to remain safe when discharged.

Likewise, a significant proportion of sexual assault survivors also experienced homelessness in a way that was related to the assault (such as being coerced into sex and assaulted in exchange for shelter or being assaulted while sleeping in a public space). This highlights the vulnerabilities to violence created by a lack of shelter as well as the importance of securing shelter after receiving hospital-based care in the wake of experiencing violence. Shelter resources for individuals who are chronically undomiciled, have psychiatric medical conditions, or substance use disorder are needed as these populations are likely simultaneously more at risk for abuse or coercion and more difficult to engage in services. Individuals experiencing both violence and substance use disorder likely need specialized intersectional resources such as treatment with buprenorphine and toxicology clinic support services while in shelters or programs.

The EDOU supportive care protocol demonstrated that safe disposition for survivors of violence is more possible with additional dedicated time and supportive effort. While provided by ED social workers in this model, such supportive care is also an integral component of patient navigation programs, which could be a complement to an EDOU supportive care protocol. With the intention of providing a patient-centered and holistic model of care, patient navigation aims to make the transition to care easier for patients by removing barriers.^23^ Patient navigation programs have shown improved health outcomes for patients, reduced unmet needs, increased self-efficacy, increased access to care, and heightened patient satisfaction. Additionally, patient navigation services improved patients’ satisfaction with healthcare clinicians, increased their communication with community services, and led to stronger care coordination.^23^

## LIMITATIONS

Efforts to improve safe disposition for IPV survivors require increased social work effort, including repeated calls to community service partners and follow-up evaluations to reassess patients. The analysis of social worker tasks did not account for the time burden that different tasks or problems require.

Because the study site serves as a rape crisis center, survivors of sexual assault routinely receive care from designated Sexual Assault Nurse Examiners with evidence collection, crisis counselor assistance, and post- exposure prophylaxis treatment for sexually transmitted disease. On occasional shifts when there is no rape crisis counselor on call, social workers provide counseling and education regarding support services. Thus, social worker encounters related to sexual assault only represent a subset of the patients evaluated at this study site following such experience. Likewise, during the review period there were also specific nurse leaders who assisted victims of sex trafficking to contact partner organizations and assist with shelter. Those who were helped by nursing did not require social work evaluation and therefore were not included in this analysis. Other patients who eloped or left before social work evaluation were also not likely recorded in the social work encounters.

This review included encounters with patients who overwhelmingly identified as female, although some male survivors were identified. This may reflect a clinical failure to adequately screen for or recognize IPV or sex trafficking in the male population. The 2020–2021 portion of this study took place during the COVID-19 pandemic. The limitations associated with this context include the strain on public resources during the pandemic, as well as the observed increase in GBV that occurred during the pandemic. This context may limit the applicability and usefulness of the proposed protocol in a non-pandemic time. Finally, this study took place in a single hospital setting; while the results are not generalizable they may inform efforts in other hospital locations.

## CONCLUSION

Survivors of gender-based violence seeking hospital-based care often have acute social support needs. In our study site social worker time was largely spent on transportation logistics with a very small proportion of encounters being explicitly tied to experiences of IPV, sexual assault or sex trafficking. A significant proportion of GBV survivors required safe housing but were unable to obtain it, placing them at risk for further violence, abuse, and exploitation. The supportive protocol of the emergency department observation unit substantially increased the proportion of GBV survivors who experienced a safe discharge.

**Summary of findings:**

- ED social work staff experience limited bandwidth to assist with navigation of accessing community-based resources for safe disposition from the emergency department following experience or disclosure of IPV and GBV.
- 40% of patients who experienced IPV and 47% who experienced sex trafficking were discharged with no safe shelter identified.
- Experience of homelessness was associated with 32% of the patients treated for sexual assault.
- Through an average 24.3 hours of an extended ED observation protocol, 70% of patients were able to achieve a safe disposition.

**Implications for practice, policy, and research:**

- Survivors of GBV who are treated in the ED have immediate need for additional safe disposition resources including additional shelter capacity.
- Enhanced services such as extended observation protocol and patient navigators would likely improve survivors’ experience of successfully accessing available community resources.
- Shelter availability would also protect survivors from further risk of GBV associated with the experience of homelessness.

## Figures and Tables

**Table 1:** Disposition survivors of intimate partner violence after hospital-based care at a public hospital in Atlanta, Georgia (2019).

Disposition	N (%)
Discharge to self	97 (39.59%)
Discharge to family/friend	69 (28.16%)
Discharge to home (safe disposition)	49 (20.00%)
Discharge to domestic violence/human trafficking shelter	14 (5.7%)
Admitted to hospital	13 (5.3%)
Discharge to homeless shelter	2 (0.82%)
Eloped	1 (0.41%)
Psychiatric admission	0 (0%)
Total	245

**Table 2:** Disposition of survivors identified with a likely experience of sex trafficking after hospital-based care in a public hospital in Atlanta, Georgia (2019).

Disposition	N (%)
Discharge to self	9 (47.40%)
Discharge to domestic violence/human trafficking shelter	2 (10.50%)
Discharge to family/friend	2 (10.50%)
Discharge to home (Safe disposition)	2 (10.50%)
Discharge to homeless shelter	2 (10.50%)
Psychiatric admission	2 (10.53%)
Total	19 (100%)

**Table 3:** Disposition of sexual assault survivors after hospital-based care in a public hospital in Atlanta, Georgia (2019).

Disposition	N (%)
Discharge to self	53 (56.38%)
Discharge to home (safe disposition)	20 (21.28%)
Discharge to family/friend	10 (10.64%)
Psychiatric admission	4 (4.26%)
Discharge to homeless shelter	3 (3.19%)
Admit	2 (2.13%)
Discharge to domestic violence/human trafficking shelter	1 (1.06%)
Eloped	1 (1.06%)
Total	94

**Table 4:** Safe disposition location for survivors of gender-based violence after participation in a hospital-based extended care protocol in a public hospital in Atlanta, Georgia (2020–2021).

Disposition location	N (%)
Discharged to family/friend	19 (32.75%)
Discharged to shelter	18 (31.03%)
Discharge to self	17 (29.31%)
Other	4 (6.89%)
Safe disposition total	41 (70.68%)

## References

[1] Houry DE, Smith SG, Chen J (2017). The National Intimate Partner and Sexual Violence Survey (NISVS) | 2010–2012 State Report The National Intimate Partner and Sexual Violence Survey (NISVS): 2010–2012 State Report. https://www.cdc.gov/violenceprevention/pdf/NISVS-StateReportBook.pdf.

[2] Centers for Disease Control and Prevention (2022). Violence Prevention: Sex Trafficking.

[3] Piquero AR, Jennings WG, Jemison E (2021). Domestic violence during the COVID-19 pandemic: evidence from a systematic review and meta-analysis. J Crim Justice.

[4] Wood L, Baumler E, Schrag RV (2022). “Don’t know where to go for help”: safety and economic needs among violence survivors during the COVID-19 Ppandemic. J Fam Violence.

[5] Jetelina KK, Knell G, Molsberry RJ (2021). Changes in intimate partner violence during the early stages of the COVID-19 pandemic in the USA. Inj Prev.

[6] al Mamun F, Hosen I, Mamun MA (2021). Sexual violence and rapes’ increment during the COVID-19 pandemic in Bangladesh. EClinicalMedicine.

[7] Coxen J, Castro V, Carr B (2021). COVID-19 pandemic’s impact on online sex advertising and sex trafficking.

[8] Rockowitz S, Stevens LM, Rockey JC (2021). Patterns of sexual violence against adults and children during the COVID-19 pandemic in Kenya: A prospective cross-sectional study. BMJ Open.

[9] Schrag RV, Leat S, Wood L (2022). “Everyone is living in the same storm, but our boats are all different”: safety and safety planning for survivors of intimate partner and sexual violence during the COVID-19 pandemic. J Interpers Violence.

[10] Williams EE, Arant KR, Leifer VP (2021). Provider perspectives on the provision of safe, equitable, trauma-informed care for intimate partner violence survivors during the COVID-19 pandemic: a qualitative study. BMC Womens Health.

[11] Roberts GL, O’Toole BI, Lawrence JM (1993). Domestic violence victims in a hospital emergency department. Med J Aust.

[12] Goldberg Pp, Moore JL, Barron CE (2019). Domestic minor sex trafficking: guidance for communicating with patients. Hosp Pediatr.

[13] Basu S, Ratcliffe G (2014). Developing a multidisciplinary approach within the ED towards domestic violence presentations. Emerg Med J.

[14] Hinsliff-Smith K, McGarry J (2017). Understanding management and support for domestic violence and abuse within emergency departments: a systematic literature review from 2000–2015. J Clin Nurs.

[15] Wood L, Schrag RV, Baumler E (2022). On the front lines of the COVID-19 pandemic: occupational experiences of the Intimate Partner Violence and Sexual Assault Workforce. J Interpers Violence.

[16] McGarry J, Nairn S (2015). An exploration of the perceptions of emergency department nursing staff towards the role of a domestic abuse nurse specialist: a qualitative study. Int Emerg Nurs.

[17] O’Doherty L, Hegarty K, Ramsay J (2015). Screening women for intimate partner violence in healthcare settings. Cochrane Database Syst Rev.

[18] Kaltiso SAO, Greenbaum VJ, Moran TP (2021). Feasibility of a screening tool for sex trafficking in an adult emergency department. Acad Emerg Med.

[19] Stoklosa H, Showalter E, Melnick A (2017). Health care providers’ experience with a protocol for the identification, treatment, and referral of human-trafficking victims. J Hum Traffick.

[20] Hackenberg EAM, Sallinen V, Handolin L (2021). Victims of severe intimate partner violence are left without advocacy intervention in primary care emergency rooms: a prospective observational study. J Interpers Violence.

[21] Egyud A, Stephens K, Swanson-Bierman B (2017). Implementation of human trafficking education and treatment algorithm in the emergency department. J Emerg Nurs.

[22] Evans DP, Hawk SR, Ripkey CE (2021). Domestic violence in Atlanta, Georgia, before and during COVID-19. Violence Gend.

[23] Valaitis RK, Carter N, Lam A (2017). Implementation and maintenance of patient navigation programs linking primary care with community-based health and social services: a scoping literature review. BMC Health Serv Res.

